# A comprehensive pan-cancer analysis of prognostic value and potential clinical implications of FTH1 in cancer immunotherapy

**DOI:** 10.1007/s00262-023-03625-x

**Published:** 2024-01-28

**Authors:** Yuejun Luo, Chengming Liu, Yuxin Yao, Xiaoya Tang, Enzhi Yin, Zhiliang Lu, Nan Sun, Jie He

**Affiliations:** 1 Department of Thoracic Surgery, National Cancer Center/National Clinical Research Center for Cancer/Cancer Hospital, Chinese Academy of Medical Sciences and Peking Union Medical College, Beijing, 100021 China; 2 State Key Laboratory of Molecular Oncology, National Cancer Center/National Clinical Research Center for Cancer/Cancer Hospital, Chinese Academy of Medical Sciences and Peking Union Medical College, Beijing, China

**Keywords:** FTH1, Immunotherapy, Single-cell, Macrophage, And TIME

## Abstract

**Background:**

Numerous studies have highlighted the crucial value of the heavy chain of ferritin (FTH1) as a key regulator of iron metabolism and a suppressor of ferroptosis, intimately tied to the tumor immune microenvironment (TIME). Nevertheless, the precise impact of FTH1 on cancer immunotherapy remains vague. Our study aims to systematically explore the prognostic significance and immune role of FTH1 in pan-cancers immunotherapy.

**Methods:**

Our study delves into the potential of FTH1 as an immunotherapeutic target within the TIME of various solid cancers. The immune landscape and underlying mechanisms of FTH1 in the TIME were investigated by multiple algorithms and bioinformatics methods. Single-cell sequencing analysis and multiplex immunofluorescence staining techniques are applied to observe FTH1 co-expression on both tumor and immune cells.

**Results:**

FTH1 exhibited aberrant expression patterns across multiple cancers, which is strongly correlated with immunotherapy resistance. Patients with high FTH1 expression levels tended to derive less benefit from immunotherapies. Moreover, FTH1 demonstrated a significant correlation with TIME infiltration, immune checkpoint molecules, and immune-related pathways. Notably, FTH1 showed a positive association with macrophage infiltrations, its expression was particularly noteworthy in malignant cells and macrophages. Inhibiting FTH1-related signaling pathways appeared to be a potential strategy to counteract tumor immunotherapy resistance.

**Conclusion:**

Our comprehensive analyses may offer valuable insights into the role of FTH1 in tumor immunotherapy. The observed correlations pave the way for further functional experiments, fostering an enhanced understanding that could shape future research endeavors.

**Supplementary Information:**

The online version contains supplementary material available at 10.1007/s00262-023-03625-x.

## Introduction

Over the past decades, our understanding of cell death has expanded with the identification of various pathways, encompassing both accidental and regulated mechanisms [1–3]. Among these, ferroptosis, a distinctive form of cell demise characterized by iron-dependent phospholipid peroxidation and reactive oxygen species, was first proposed by Professor Dixon [4, 5]. This unique mode of cell death, distinguished by specific morphological features and molecular intricacies, has been implicated in a variety of degenerative diseases, particularly in the context of tumorigenesis and progression [6–8]. Notably, ferroptosis not only triggers a robust tumor immune response through the release of damage-related molecular patterns but also collaborates with various antitumor agents to impede tumor growth [9, 10], with key regulators of ferroptosis playing pivotal roles in these processes.

Ferritin, serving as a crucial iron storage form in mammals, mediates iron homeostasis across diverse pathophysiological processes, especially in the ferroptosis of cancers [11–15]. The heavy chain of ferritin (FTH1) serves as the key modulator in regulating the iron metabolism (Fenton reaction) in ferroptosis, and is concurrently identified as a critical suppressor of this process in numerous cancers [16, 17]. FTH1 is able to upregulate the iron storage, which decreasing the level of cellular Fe^2+^ (fuel of ferroptosis) and impairing the ferroptosis activation pathways. Overexpressing FTH1 will be a significant inhibition of the cellular ferroptosis [18]. Mounting evidence underscores the role of aberrant iron metabolism not only in influencing tumor growth but also in shaping alterations in tumor immune microenvironment (TIME) infiltration, ultimately impacting the efficacy of immunotherapy [14, 15, 19, 20]. Given its significance as an iron metabolism regulator and ferroptosis suppressor, FTH1 may intricately link with the tumor immune microenvironment and responses to immunotherapy. While previous studies have offered preliminary insights into the role of FTH1 in specific cancers [21, 22], its broader implications in the realm of immunotherapy across diverse cancers remain unknown. A comprehensive analysis of the association between FTH1 levels and immunotherapy responses across various cancers is warranted to enhance our understanding of the impact of ferroptosis on immunotherapy.

This study systematically investigated the significance of FTH1 in immunotherapy responses within the context of a multitude of solid cancers. Meanwhile, we explored the relationship between FTH1 and immunotherapy resistance, initially substantiating its close association with the suppressive TIME. Employing single-cell sequencing analysis and multiplex immunofluorescence staining, we elucidated the expression landscape of FTH1 within the TIME. The results of our study may offer some novel insights into the value of FTH1 in diverse tumors, particularly its therapeutic potential in immunotherapy, laying the groundwork for further functional experiments in the future.

## Materials and methods

### Collection of samples

Genomic, clinical, and somatic mutation data for 33 solid cancers were obtained from The cancer genome atlas (TCGA) database (https://portal.gdc.cancer.gov/). Five immunotherapeutic cohorts, encompassing GSE67501 (patients with renal cell carcinoma accepting nivolumab treatment), GSE100797 (patients with melanoma accepting adoptive T cell therapy), GSE115821 (patients with metastatic melanoma accepting anti-PD-1/CTLA4 treatment), GSE145996 (patients with metastatic melanoma accepting anti-PD-1 treatment), and GSE173839 (patients with breast cancer accepting durvalumab treatment), were enrolled. Expression profiles and clinical information for these cohorts can be downloaded from the gene expression omnibus (GEO) database (http://www.ncbi.nlm.nih.gov/geo). Cell line data were sourced from the cancer cell line encyclopedia (CCLE) website (http://www.sites.broadinstitute.org/ccle). Single-cell sequencing datasets for cervical cancer (GSE171894), esophageal squamous carcinoma (GSE188900), hepatocellular carcinoma (GSE125449), and gastric cancer (GSE183904) were included.

### Protein–protein interaction (PPI) network analysis

The STRING website (http://string-db.org/cgi/input.pl) was utilized for the protein–protein interaction network analysis of FTH1-related genes [23], and the Cytoscape software was employed for the visualization of corresponding results.

### Tumor immune dysfunction and exclusion (TIDE) analysis

TIDE is a classical computational framework to predict immune checkpoints blockade treatment responses in cancers [24]. The TIDE score is regarded as a reliable biomarker in predicting immunotherapy responses in patients accepting anti-PD-1/L1 or anti-CTLA drugs, especially in lung cancer and melanoma. The transcriptome profiles of the various cancers were uploaded to the TIDE website (http://tide.dfci.harvard.edu), and after online analysis based on the tools of this website, the corresponding TIDE scores for all patients were downloaded for subsequent analysis.

### Identification of immune characteristics

ESTIMATE is a tool used to analyze tumor purity and the infiltration of immune and stromal cells in tumor tissues [16]. We used the ESTIMATE algorithm (ESTIMATE package) to calculate three scores for every sample: (1) tumor purity score, (2) immune score (predicts the presence of immune cells), and (3) stromal score (represents the infiltration of stromal cells). The TIMER, EPIC, QUANTISEQ, and MCP-COUNTER algorithms were applied to analyze the relationship between the FTH1 expression level and the infiltration of various immune cells.

#### GSEA

GSEA was conducted to explore signaling pathways differences between low and high FTH1 expression groups using Kyoto encyclopedia of genes and genomes (KEGG) terms. The R software (version 3.5.3) and the limma, org.Hs.eg.db, clusterProfiler, enrichplot, and DOSE packages were used for analysis and visualization.

### Analysis of FTH1 expression and dynamic immunologic features (TMB and MSI)

Tumor mutation burden (TMB) was calculated as the total number of genetic mutations in cancer cells, including errors in somatic gene coding, gene insertions, gene deletions, and base substitutions, and then the total number of mutations was divided by the exome size. We calculated the TMB of every tumor sample, utilizing a standardized exome size of 38 Mb. The microsatellite instability (MSI) scores of the TCGA samples were obtained from previously published research materials [24]. Associations between FTH1 expression and TMB/MSI were examined.

### Multiplex immunofluorescence staining

The formalin-fixed, paraffin-embedded pan-cancer tissues microarrays (TMAs) were purchased from Shanghai Outdo Biotech Company. The slides were firstly deparaffinized, rehydrated in pure ethanol and distilled water sequentially, and then incubated with 3% H_2_O_2_ for 25 min in the dark to block endogenous peroxidase activity. The phosphate-buffered saline plus 2% bovine serum albumin was used to dilute the primary and second Abs. The primary Abs includes CD68 (1:300 dilution, 25747-1-AP, Proteintech), CD163 (1:3000 dilution, 68218-1-Ig, Proteintech), and FTH1 (1:1000 dilution, ab65080, Abcam). The fluorescence spectra were captured from 420 to 720 nm with the same exposure time. The Caseviewer software was applied to analyze the multispectral images.

### Single-cell sequencing analysis

Quality control of the analyzed data was performed based on the Python package Scanny. The principal component analysis was used for dimension reduction. Batch effects were removed using the Harmony R package. We visualized the dimensionality reduction via the UMAP function, and all cells were clustered using the Leiden algorithm. Vlnplot, Dimplot, and Featureplot methods were used for comprehensive visualization of FTH1 expression details.

### Analysis of immunotherapeutic responses

The immunotherapeutic responses were measured and assessed according to the RECIST V1.1 Criteria: complete response (CR), partial response (PR), stable disease (SD), and progressive disease (PD). CR and PR patients were assigned to the response group, and SD and PD patients were assigned to the non-response group. The student’s t test method was applied to investigate the difference in FTH1 expression between the two groups.

### Statistical analysis

Prognostic significance of FTH1 was explored by the log-rank test across the 33 tumors based on the optimal cutoff value. The *P* value < 0.05 was regarded as statistically significant. All tests involved in this research were two-sided.

## Results

### FTH1 mRNA expression in most human cancers

**Fig. 1:**
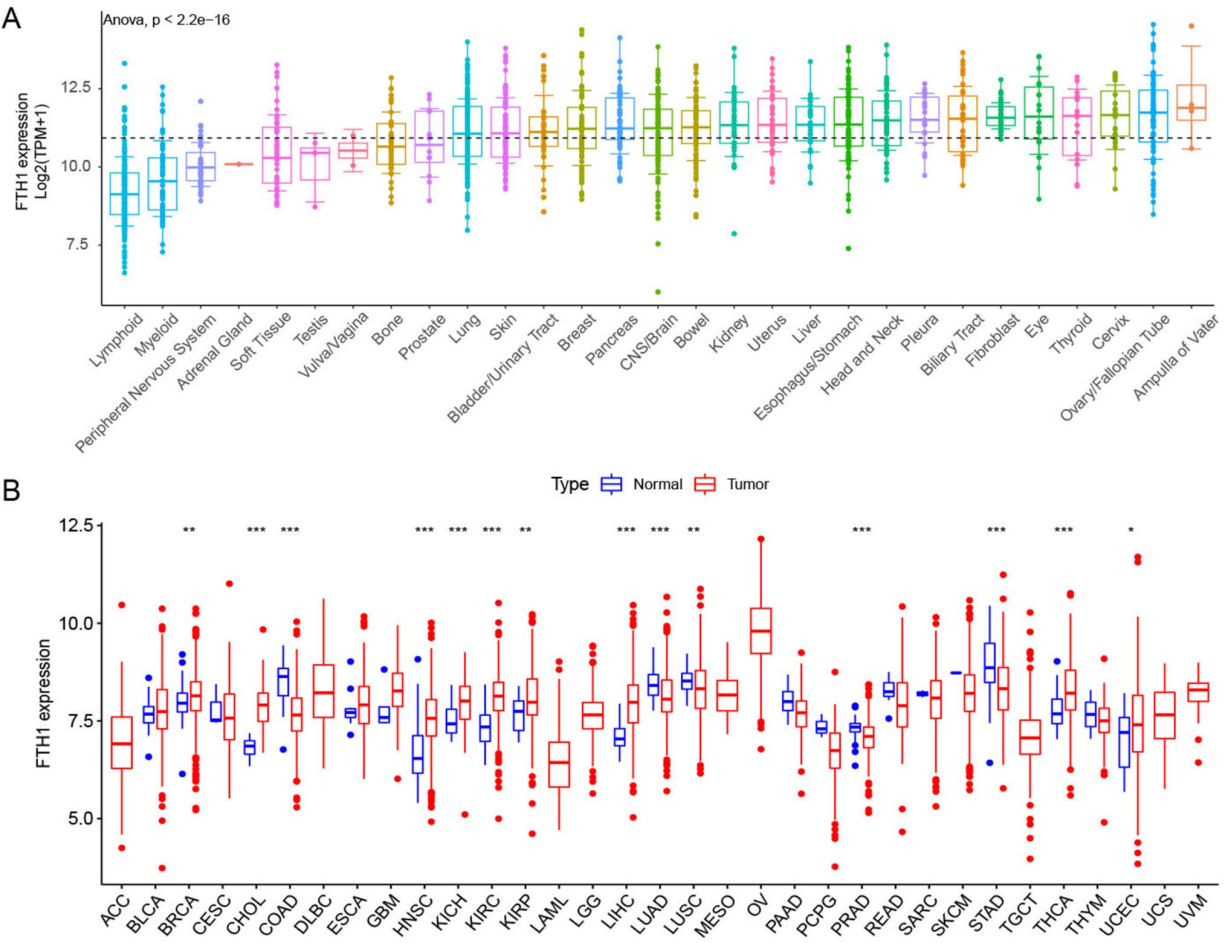
Expression landscape of FTH1 in the normal and tumor tissues. **A** FTH1 expression levels in tumor cell lines from the CCLE dataset. **B** FTH1 expression in the normal and tumor samples analyzed by TCGA dataset

To present a comprehensive overview, a flowchart of the analysis is displayed in Fig. S1, with 33 tumors abbreviations listed in Table 1. Firstly, to comprehensively exhibit the expression details of FTH1 in tumor and adjacent samples, we found high FTH1 expression in various tumor cell lines, particularly in the ampulla of Vater, ovary, cervix, and thyroid according to the CCLE dataset (Fig. 1A). Additionally, we identified the top 100 FTH1-related genes from the CCLE dataset, and the corresponding PPI analysis is shown in Fig. S2. Analyzing tumor and normal samples from the TCGA dataset revealed significant upregulation of FTH1 in 10 tumors (BRCA, ESCA, KIRP, UCEC, HNSC, KIRC, LIHC, THCA, KICH, CHOL) and slight downregulation in LUAD, COAD, PRAD, LUSC, and READ, compared to normal tissue (Fig. 1B). We also ranked the expression level of FTH1 in various tumors (Fig. S3). Combined with the results of the CCLE database described above, we find a consistency in the analysis of the two databases. FTH1 was highly expressed in OV and THCA, while lowly expressed in ACC and LAML, which is consistent with the findings in the CCLE database (high expression in the ampulla of Vater and thyroid, low expression in adrenal gland and lymphoid) (Table 1).

**Table 1:** The 33 types of human cancers enrolled in this study

Abbreviation	Full name
ACC	Adrenocortical carcinoma
BLCA	Bladder urothelial carcinoma
BRCA	Breast invasive carcinoma
CESC	Cervical squamous cell carcinoma and endocervical adenocarcinoma
CHOL	Cholangiocarcinoma
COAD	Colon adenocarcinoma
DLBC	Lymphoid neoplasm diffuse large B-cell lymphoma
ESCA	Esophageal carcinoma
GBM	Glioblastoma multiforme
HNSC	Head and neck squamous cell carcinoma
KICH	Kidney chromophobe
KIRC	Kidney renal clear cell carcinoma
KIRP	Kidney renal papillary cell carcinoma
LAML	Acute myeloid leukemia
LGG	Brain lower grade glioma
LIHC	Liver hepatocellular carcinoma
LUAD	Lung adenocarcinoma
LUSC	Lung squamous cell carcinoma
MESO	Mesothelioma
OV	Ovarian serous cystadenocarcinoma
PAAD	Pancreatic adenocarcinoma
PCPG	Pheochromocytoma and paraganglioma
PRAD	Prostate adenocarcinoma
READ	Rectum adenocarcinoma
SARC	Sarcoma
SKCM	Skin cutaneous melanoma
STAD	Stomach adenocarcinoma
TGCT	Testicular germ cell tumors
THCA	Thyroid carcinoma
THYM	Thymoma
UCEC	Uterine corpus endometrial carcinoma
UCS	Uterine carcinosarcoma
UVM	Uveal melanoma

### Immunotherapeutic responses predictions of FTH1 expression

**Fig. 2:**
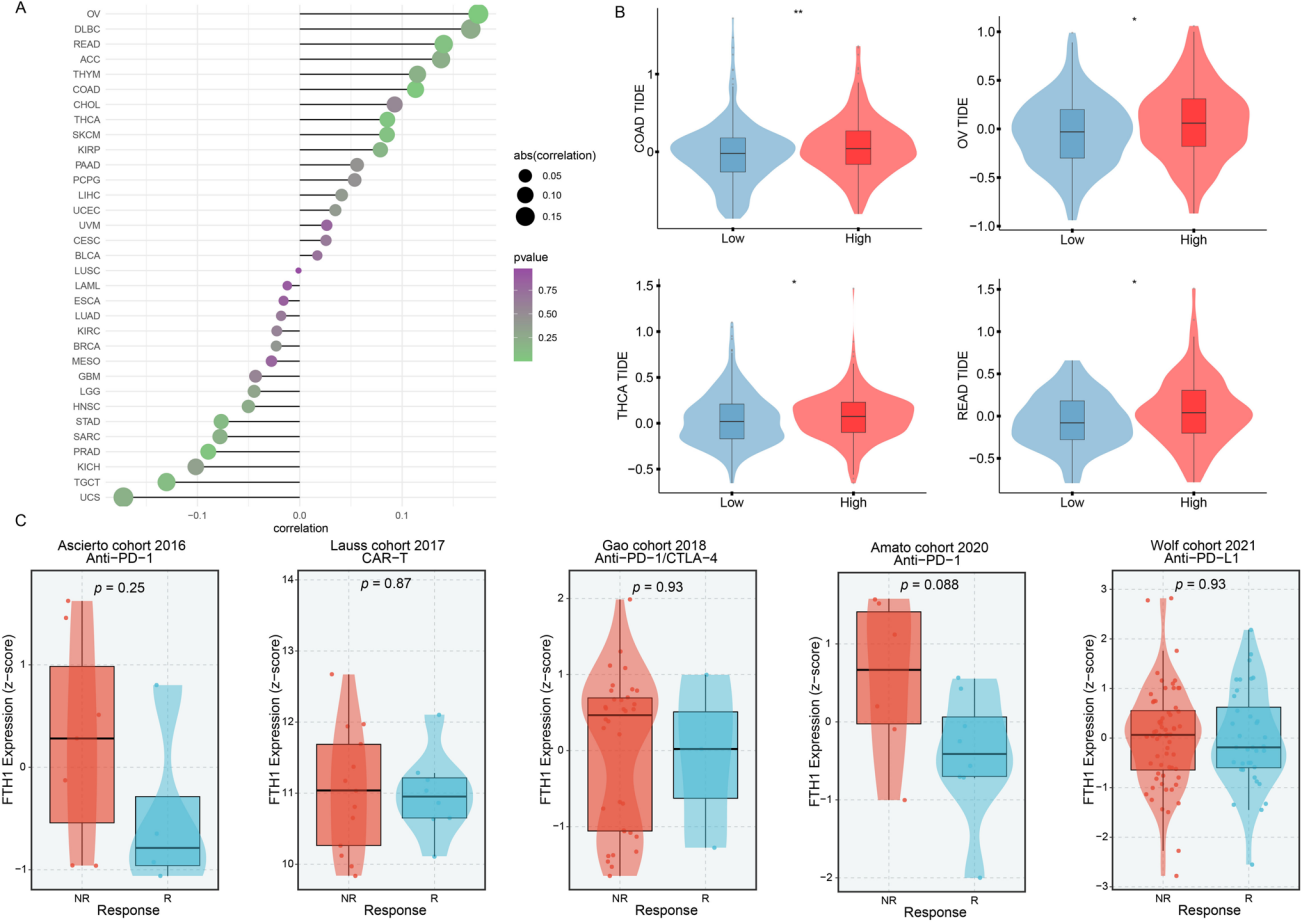
The association of FTH1 expression with immunotherapy response and Tumor Immune Dysfunction and Exclusion (TIDE) scores. **A** The association between FTH1 expression and TIDE score. **B** The distribution of TIDE scores across FTH1 high and low expression groups in various tumors. **C** The expression of FTH1 in response and non-response groups of different immunotherapeutic cohorts

To investigate the potential role of FTH1 in pan-cancer immunotherapy, we tried to calculate the TIDE score, which is a classical and reliable biomarker for immunotherapy responses, in patients from different expression groups. TIDE scores exhibited a positive correlation with FTH1 expression levels in most solid malignancies, especially in OV, READ, COAD, and THCA (Fig. 2A). Patients with high FTH1 expression showed higher TIDE scores than low expression counterparts in some solid cancers, suggesting potential reduced benefits from immunotherapy (Fig. 2B). Furthermore, we investigated FTH1 in various immunotherapy cohorts, including anti-PD1/L1, anti-CTLA4, and CAR-T treatments, revealing higher FTH1 expression in the non-response group in comparison to the response group (Fig. 2C). Collectively, we speculated that high FTH1 expression may be closely related to immunotherapy resistance.

### Prognostic value of FTH1

**Fig. 3:**
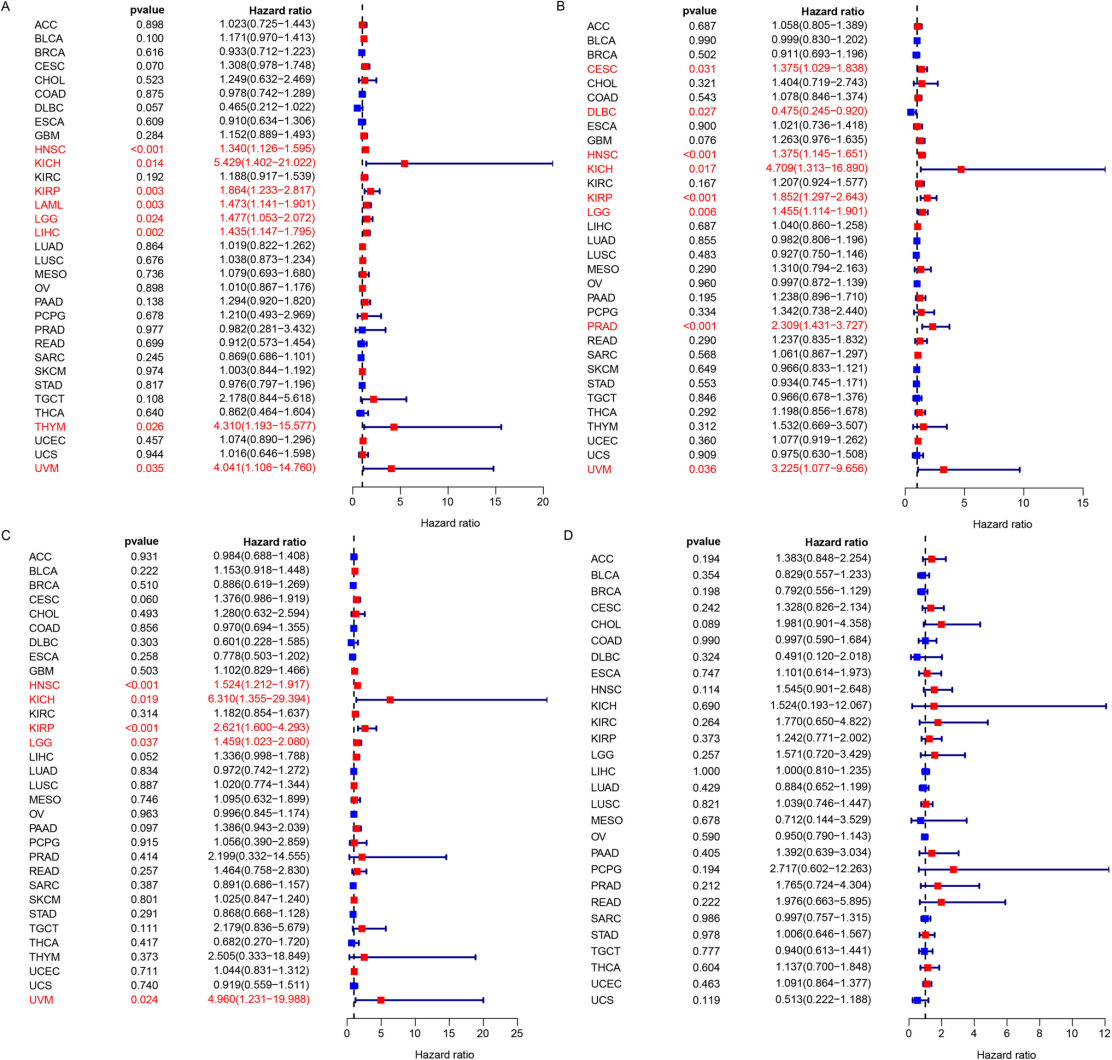
Survival analysis of FTH1 expression from the TCGA database. **A** Forest plot of survival analysis of FTH1 in OS. **B** Forest plot of survival analysis of FTH1 in PFS. **C** Forest plot of survival analysis of FTH1 in DSS. **D** Forest plot of survival analysis of FTH1 in DFS

We evaluated the impact of aberrant FTH1 mRNA expression on various cancers using the Kaplan–Meier plotter. TCGA analysis indicated that FTH1 played unfavorable prognostic roles in different cancers. The univariate cox regression analysis exhibited that high FTH1 level was related to unfavorable overall survival (OS) in HNSC, KICH, KIRP, LAML, LGG, and LIHC (Fig. 3A). Upregulated FTH1 expression predicted shorter progression-free survival (RFS) in CESC, HNSC, KICH, KIRP, LGG, PRAD, and UVM (Fig. 3B). Additionally, high FTH1 expression was related to poor disease-specific survival (DSS) in HNSC, KICH, KIRP, LGG, and UVM (Fig. 3C). The OS analysis of FTH1 based on the most optimal cut-off point in most cancers was shown in Fig. S4–S5. Furthermore, we also performed the ROC analysis in the 5-year OS, we found that FTH1 expression level exhibited a relatively well-predicted prognostic value in various tumors, especially in CESC (cut-off value, 7.358), KICH (cut-off value, 8.0270), KIRP (cut-off value, 8.1486), LAML (cut-off value, 6.5057), LGG (cut-off value, 8.0822), THYM (cut-off value, 7.8515), and UVM (cut-off value, 8.0819), with AUC values of 0.579, 0.776, 0.562, 0.685, 0.586, 0.772, and 0.728, respectively (Fig. S6).

### Immune aspects of FTH1 in the tumor immune microenvironment

**Fig. 4:**
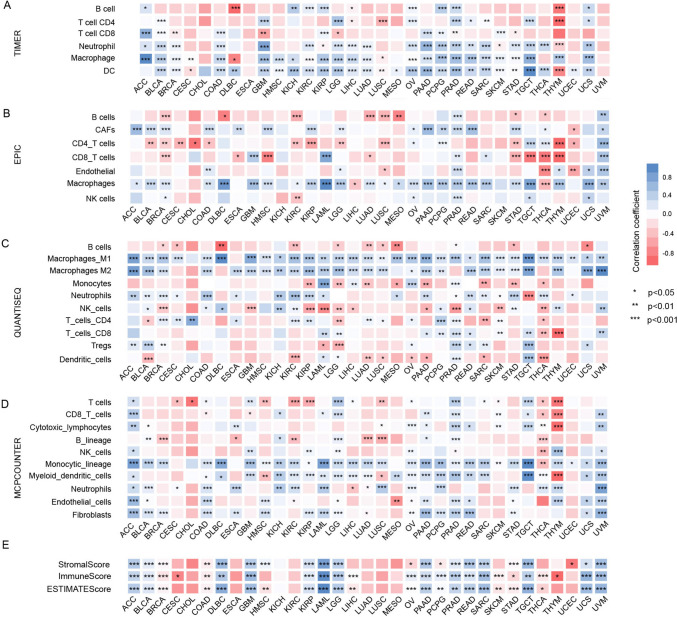
Relationship between FTH1 expression level and tumor immune infiltrates. **A** Immune cell infiltration explored by the TIMER algorithms. **B** Immune cell infiltration explored by the EPIC algorithms. **C** Immune cell infiltration explored by the MCP-counter algorithms. **D** Immune cell infiltration explored by the CIBERSORT algorithms. **E** Correlation analysis between FTH1 and ESTIMATE, Immune, and Stromal Scores by ESTIMATE algorithms

To reveal the relationship between FTH1 and immunotherapy resistance, we analyzed various immune aspects of FTH1 in TIME. The ESTIMATE algorithm was utilized to analyze the correlation between FTH1 level and the stromal scores, immune scores, and estimate scores in various tumors. The top five cancers with a positive correlation between FTH1 level and stromal scores were LAML, UVM, DLBC, LGG, and TGCT; the top five malignancies with a remarkable relationship between FTH1 and immune score were LAML, UVM, UCS, LGG, and GBM; the top five cancers with the positive relationship between FTH1 levels and estimate scores refer to LAML, UCS, UBM, DLBC, and TGCT (Fig. 4E). Additionally, we performed four algorithms to quantify the relationship between FTH1 level and multiple immune cell infiltrations, namely TIMER, EPIC, QUANTISEQ, and MCPCOUNTER. In general, a relatively high CD8^+^ T-cell infiltration in the tumor microenvironment is often defined as a hot tumor and vice versa as a cold tumor. We observed that FTH1 exhibited different relationship with CD8^+^T cells infiltration. Combining the results of multiple algorithms in a comprehensive analysis, we found that FTH1 expression was positively correlated with CD8^+^ T cells infiltration in ACC, LAML, PAAD, PCPG, PRAD, TGCT, and UVM tumors. However, FTH1 was negatively related to CD8^+^ T cells in GBM, STAD, THCA, and THYM. Strikingly, upregulated FTH1 expression displayed a significantly positive correlation with macrophage infiltration in most cancers, especially M2 macrophages in most cancers, including ACC, BLCA, BRCA, COAD, GBM, HNSC, KICH, KIRP, LGG, LIHC, LUAD, PAAD, PCPG, PRAD, READ, TGCT, UCS, and LAML (Fig. 4A–D). Besides, elevated FTH1 level is negatively related to B cell infiltration in DLBC, THYM, KIRC, LUAD, LUSC, MESO, STAD, and THCA (Fig. 4A–D).

**Fig. 5:**
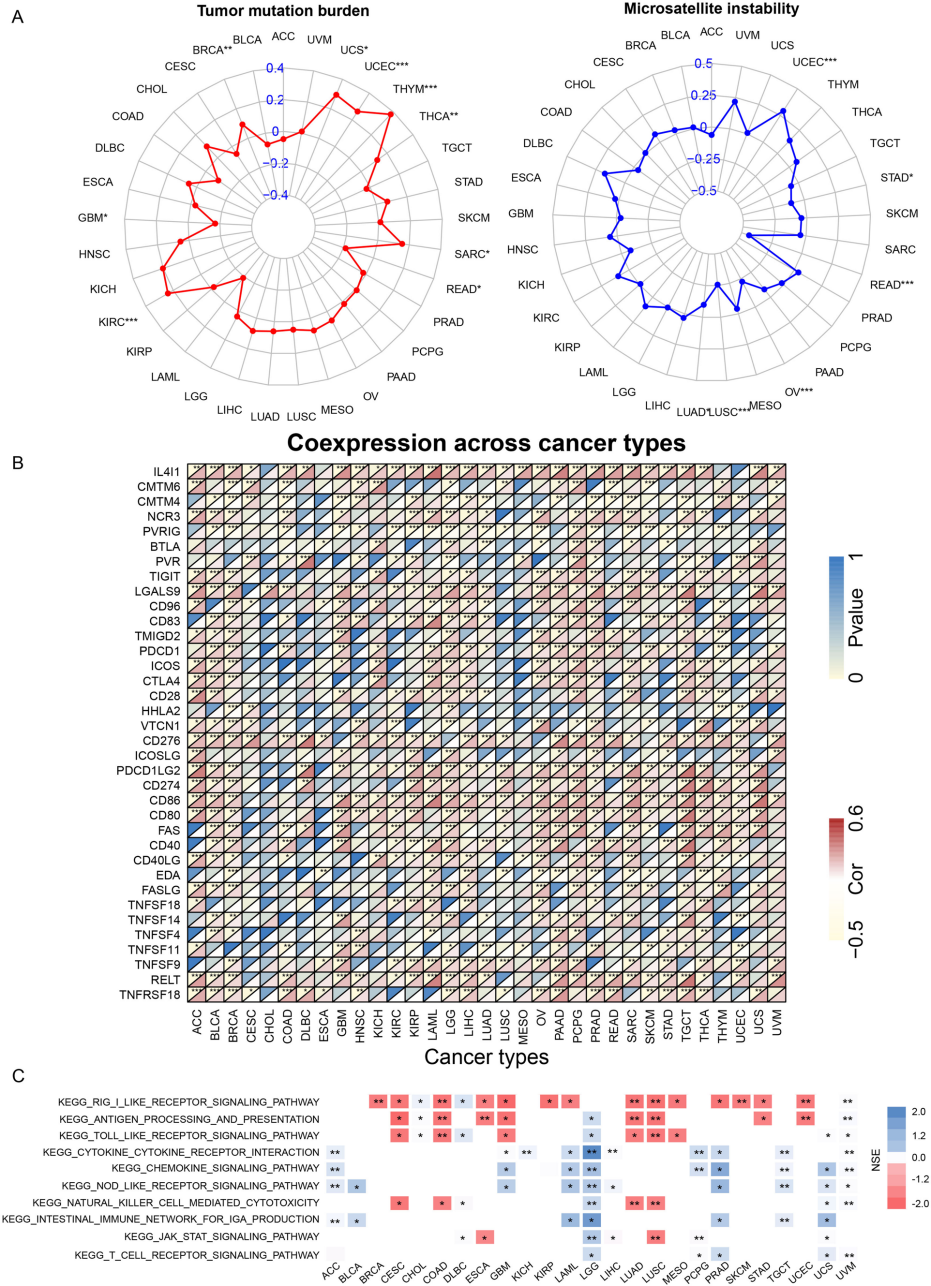
Relationship between FTH1 expression and various immune characteristics and immune-related pathways. **A** Correlation between FTH1 expression and TMB displayed by the radar chart. **B** Correlation between FTH1 expression and MSI displayed by the radar chart. **C** Relationship between FTH1 expression and various immune checkpoints. **D** Top 10 enriched immune-related pathways based on the KEGG terms

We further probed into the correlation between FTH1 and dynamic immune-related features, including TMB and MSI, the leading immunotherapeutic biomarkers. We found that FTH1 had a positive association with TMB in BRCA, KIRC, SARC, THCA, THYM, UCEC, and UCS but negatively correlated with GBM and READ (Fig. 5A). FTH1 was also demonstrated to be positively related to MSI in UCEC but negatively correlated with MSI in LUAD, LUSC, OV, READ, and STAD (Fig. 5A).

Considering the robust correlation with immune cells, we explored FTH1’s association with immunomodulatory molecules, including the B7-CD28 family, tumor necrosis factors (TNF) family, and other classic and novel immune checkpoints. The results demonstrated that these immune checkpoints are closely related to FTH1 expression in different cancers, namely PDCD1, CTLA4, CD274, and TIGIT exhibited a positive association with FTH1 in ACC, BCLA, BRCA, DLBC, KIRP, LAML, LGG, LUSC, OV, PCPG, PRAD, SARC, SKCM, TGCT, THCA, THYM, UCEC, and UCS (Fig. 5B). Most of these inhibitors have been identified as the key effectors of immunotherapy responses and novel immunotherapeutic targets. It is reasonable to speculate that FTH1 acts an indispensable function in the TIME in various tumors.

### Signaling pathways significantly influenced by FTH1

To investigate deeply the underlying mechanisms involved in the function of FTH1, we conducted KEGG GSEA across 33 human cancers. Several immune-related pathways were enriched in the high FTH1 expression group in multiple tumors. FTH1 was remarkably involved in the RIG-I-like receptor signaling, antigen process and presentation, and toll-like receptor signaling pathways in most tumors (Fig. 5C). FTH1 was also significantly related to the cytokine-cytokine receptor interaction and the chemokine signaling pathway in ACC, GBM, KICH, LAML, LGG, PCPR, PRAD, TGCT, and UVM (Fig. 5C). Furthermore, the JAK-STAT signaling pathway was enriched in the low FTH1 expression groups of the ESCA and LUSC (Fig. 5C). These results further demonstrated that FTH1 is involved in multiple immune activities and may play an indispensable role in the anti-tumor immune process.

### Single-cell sequencing and multiplex immunofluorescence staining of FTH1 in various tumors

**Fig. 6:**
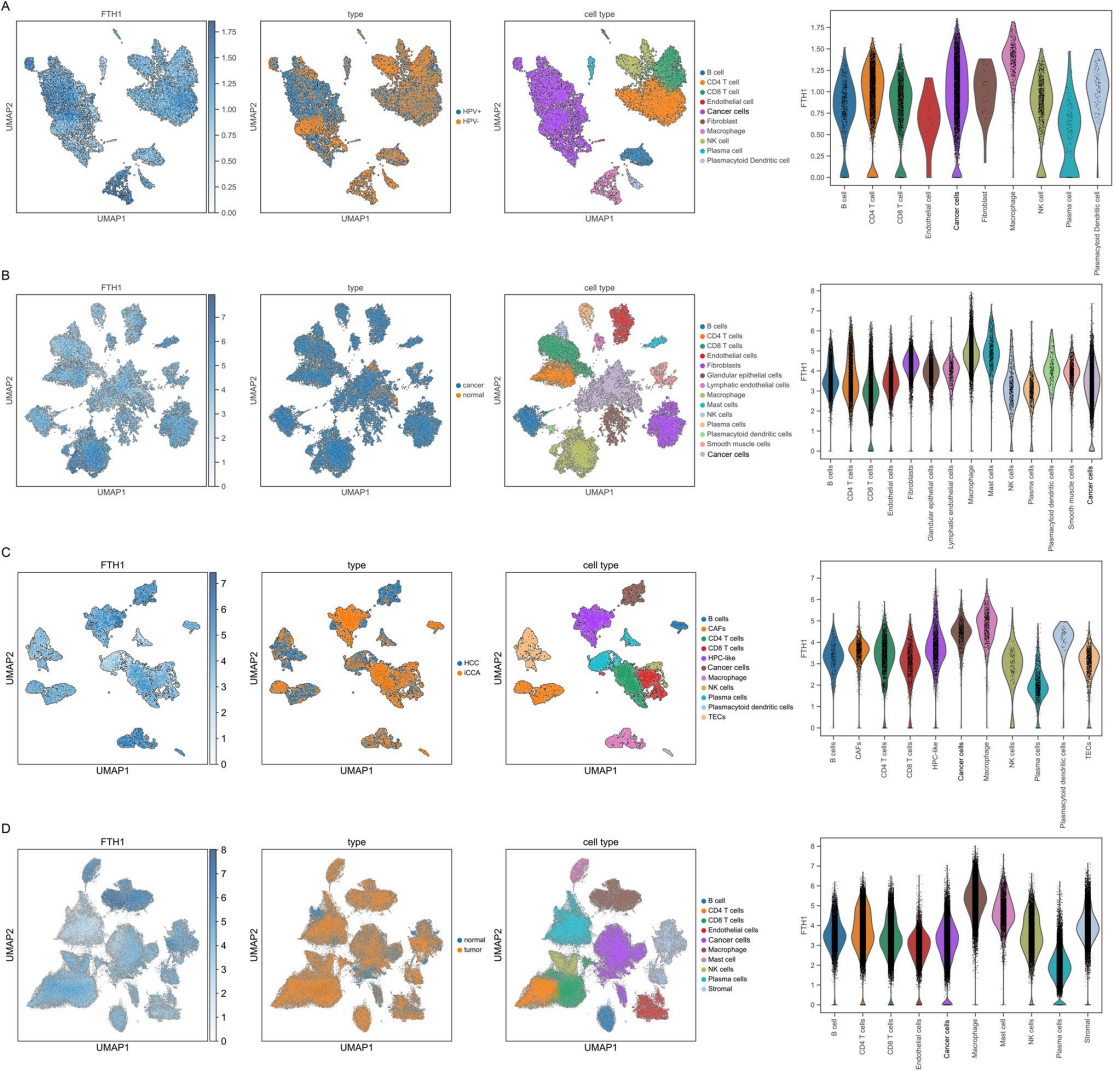
Single cell sequencing analysis of FTH1 co-expression on tumor and stromal cells in ESCC (**A**), HCC (**B**), LUSC (**C**), STAD (**D**), COAD (**E**), and THCA (**F**). CD68 was marked red, CD163 was marked green, and FTH1 was marked purple

**Fig. 7:**
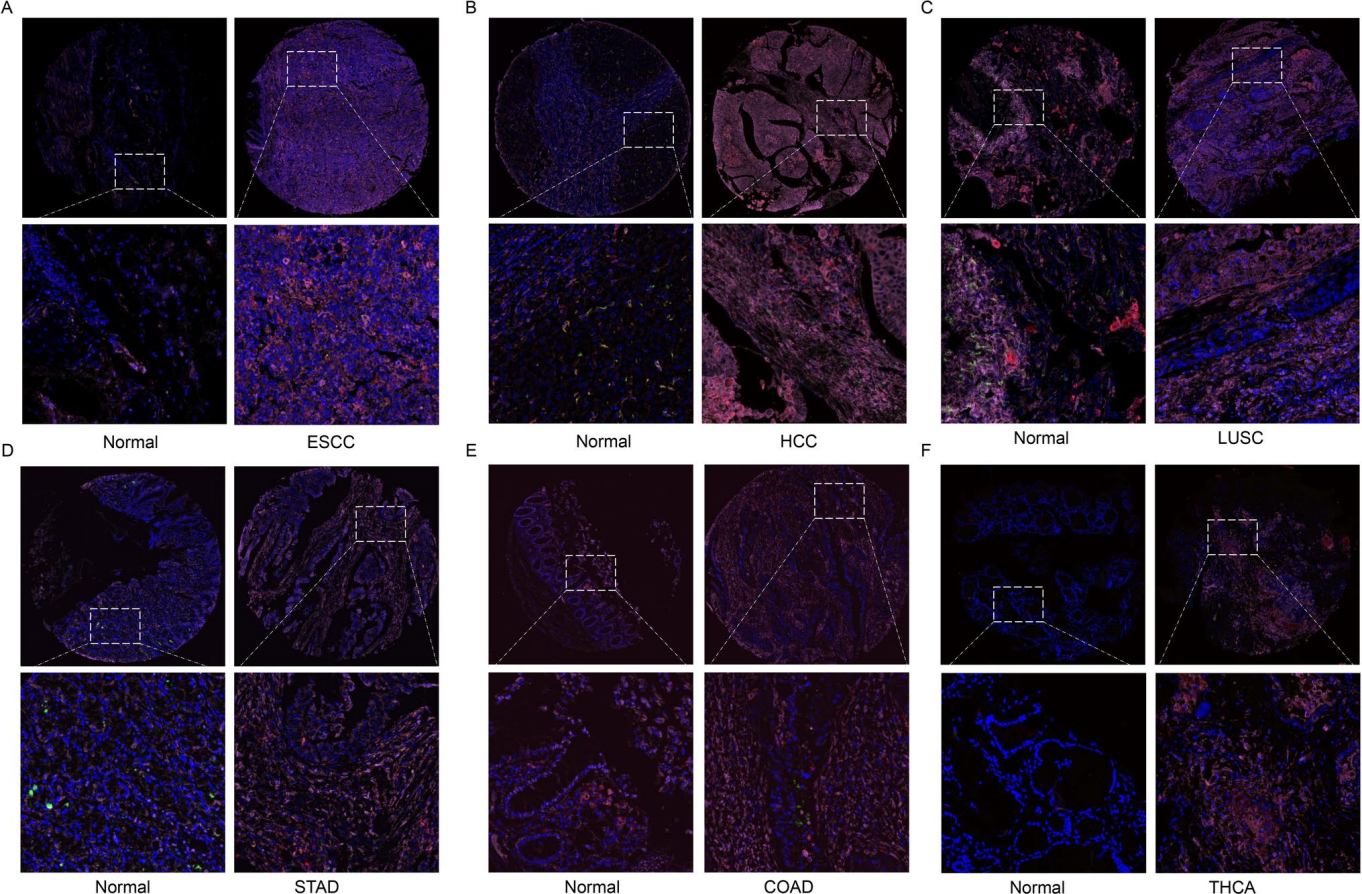
Multiplex immunofluorescence staining exploring FTH1 co-expression on M1 and M2 macrophages in various solid tumor samples

Next, we unveiled the FTH1 expression in tumor cells and various immune and stromal cells in several solid cancer types, namely CESC, ESCC, HCC, and GC (Fig. 6A–D). It is obvious that FTH1 is significantly co-expressed in tumor cells and immune and stromal cells. We observed that FTH1 exhibited higher expression level in the macrophages and tumor cells. Meanwhile, considering that FTH1 expression levels have previously demonstrated a very strong correlation with macrophage infiltrates in the vast majority of solid tumors. Furthermore, we tried to verify the co-expression of FTH1 with macrophages (M1 and M2 macrophages) in multiple cancers by multiplex immunofluorescence staining method. We observed that FTH1 was increased in tumors than in normal tissues, namely ESCC, HCC, STAD, and THCA (Fig. 7). We also found that FTH1 was highly expressed in M1 macrophages and tumor cells in the above cancers. Meanwhile, FTH1 was also expressed in CD163^+^ M2 macrophages in ESCC and HCC.

## Discussion

Substantial studies have recognized that iron metabolism acts an indispensable role in cancer biology and tumor immunity, especially the role of key regulators of Fenton reaction in various biological processes [25–27]. FTH1, a key mediator of iron metabolism, binds to transferrin receptor 1, facilitating Fe^2+^ storage in the cytoplasm and triggering iron-dependent ferroptosis. FTH1 exhibited aberrant expression profiles in most solid cancers, which may cause iron metabolism disturbance to influence cancer cell proliferation and tumor microenvironment. While previous studies have touched upon the role of FTH1 in specific cancers [21, 28], our investigation is the first to comprehensively explore the relationship between FTH1 levels and immunotherapy response in pan-cancer. This exploration enhances our understanding of the impact of ferroptosis on immunotherapy.

This time, we comprehensively investigated the value of FTH1 as a potential immunotherapeutic target candidate in the TIME among various solid cancers. We observed that patients with high FTH1 expression appeared to benefit less from various immunotherapies, including anti-PD1/L1, anti-CTLA4, and CAR-T treatments. Additionally, we assessed the association between FTH1 and classical immunotherapy biomarkers, such as TMB, MSI, and TIDE scores. We found that FTH1 had a positive correlation with TMB in most tumors, including BRCA, KIRC, SARC, THCA, THYM, UCEC, and UCS, while with a negative association in GBM and READ. TIDE score is currently a potential predictor of immunotherapy response, with increasing studies exhibiting a reliable accuracy of TIDE score in predicting the survival of patients who receive immunotherapy. We found that patients with high FTH1 expression displayed high TIDE scores in most cancers, which may benefit less from immunotherapy treatments. Taken together, a potential link may exist between high FTH1 expression and immunotherapy resistance.

To unveil the immune aspects of FTH1 in the TIME, our study demonstrated a close relationship between FTH1 levels and stromal score, immune score, and ESTIMATE score. Importantly, FTH1 was positively associated with various immune cells infiltrations, especially M2 macrophages in the most tumors, which may also be a reason why dysregulated FTH1 expression is associated with immunotherapy resistance. High FTH1 expression was associated with higher macrophage infiltration, and as reported, tumor-associated macrophages can accelerate tumor growth, progression, and resistance to therapies, including immunotherapy [29, 30]. Additionally, we evaluated the relationship between FTH1 and various novel immune checkpoints, finding positive correlations in most solid cancers, including key effectors of immunotherapy responses like PDCD1, CTLA4, CD274, and TIGIT. This implies a potential indispensable role of FTH1 in the TIME across various tumors.

Exploring the underlying molecular mechanisms of FTH1 in tumors through KEGG analyses, our results indicated significant involvement in immune-related pathways such as cytokine-cytokine receptor interaction and the chemokine signaling pathway in ACC, GBM, KICH, LAML, LGG, PCPR, PRAD, TGCT, and UV. Furthermore, the JAK-STAT signaling pathway was enriched in the low FTH1 expression groups of ESCA and LUSC, emphasizing FTH1’s role in multiple immune activities and its potential indispensable function in the anti-tumor immune process.

Furthermore, we also investigated the expression landscape of FTH1 in the TIME by exploring the expression details of diverse cell types, namely malignant cells, stromal cells, and immune cells in multiple cancers. We found that FTH1 was highly expressed in tumor cells and macrophages. We also verified the expression of FTH1 on various macrophages and tumor cells by multiplex immunofluorescence staining. We observed that FTH1 was increased in tumors than in normal tissues, including ESCC, HCC, STAD, and THCA. FTH1 was highly expressed in M1 macrophages and tumor cells in various cancers, which was also expressed in CD163^+^ M2 macrophages in ESCC and HCC. All these findings highlight a strong correlation between FTH1, cancer cells, and immune cells in TIME.

For all we know, this research is the first to systematically investigate the role of FTH1 in tumor immunology from a pan-cancer perspective. Our findings offer some valuable insights into the potential of FTH1 in cancer immunotherapy, offering a preliminary analysis of its association with immune cell infiltration, immune molecules, and classical immunotherapeutic markers. The critical role of FTH1 in tumor immunity underscores its potential as a therapeutic target to enhance the benefits of immunotherapy for a broader patient population. Importantly, we also revealed the expression details of FTH1 in TIME in various tumors for the first time. While our bioinformatics methods have shed light on these associations, further basic and clinical studies are crucial to fully elucidate the mechanisms of FTH1 in tumor immunity.

### Supplementary Information

Below is the link to the electronic supplementary material.

Supplementary file 1. Fig. S1 The flowchart of this study.

Supplementary file 2. Fig. S2 The details of protein-protein interaction network of FTH1-related genes.

Supplementary file 3. Fig. S3 The mean expression of FTH1 in different cancers (from high to low).

Supplementary file 4. Fig. S4 The Kaplan-Meier curves of OS in BLCA(A), BRCA (B), CESC (C), DLBC (D), HNSC (E), KICH (F), KIRC (G), KIRP (H), LAML (I), LGG (J), LIHC (K), and LUSC (L).

Supplementary file 5. Fig. S5 The Kaplan-Meier curves of OS in PAAD (A), SARC (B), SKCM (C), TGCT (D), THYM (E), UCEC (F), UCS (G), and UVM (H).

Supplementary file 6. Fig. S6 ROC analysis of FTH1 expression for 5-year OS in CSEC (A), KICH (B), KIRP (C), LAML (D), LGG (E), THYM (F), and UVM (G).

## Data Availability

The datasets used and analyzed during the current study are available from the corresponding author on reasonable request.

## Abbreviations

CCLE: Cancer cell line encyclopedia
CR: Complete response
DFS: Disease-free survival
DSS: Disease specific survival
GEO: Gene expression omnibus
FTH1: Heavy chain of ferritin
HPC-like: Hepatic progenitor cells
KEGG: Kyoto encyclopedia of genes and genomes
MSI: Microsatellite instability
OS: Overall survival
PD: Progressive disease
PPI: Protein–protein interaction
PR: Partial response
RFS: Progression-free survival
SD: Stable disease
TCGA: The cancer genome atlas
TECs: Tumor-associated endothelial cells
TIDE: Tumor immune dysfunction and exclusion
TIME: Tumor immune microenvironment
TMAs: Tissue microarrays
TMB: Tumor mutation burden
