# Supplementary figures and images for: A comprehensive pan-cancer analysis of prognostic value and potential clinical implications of FTH1 in cancer immunotherapy
Source: Cancer Immunol Immunother. 2024 Jan 28;73(2):37. doi: 10.1007/s00262-023-03625-x (PMC10822802; doi:10.1007/s00262-023-03625-x)

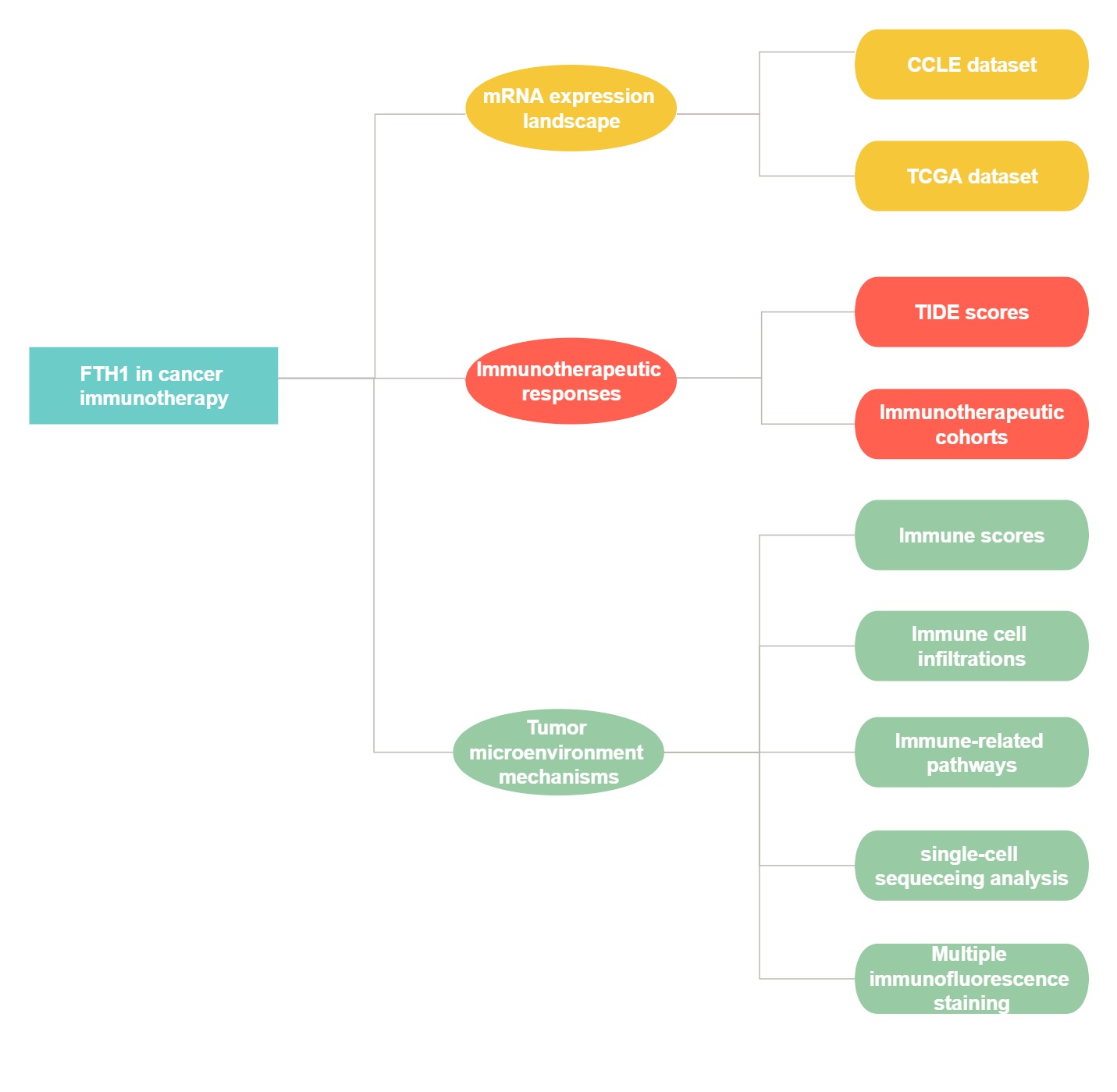

Supplement: Supplementary file 1 — Fig. S1 The flowchart of this study. [file 262_2023_3625_MOESM1_ESM.jpg]

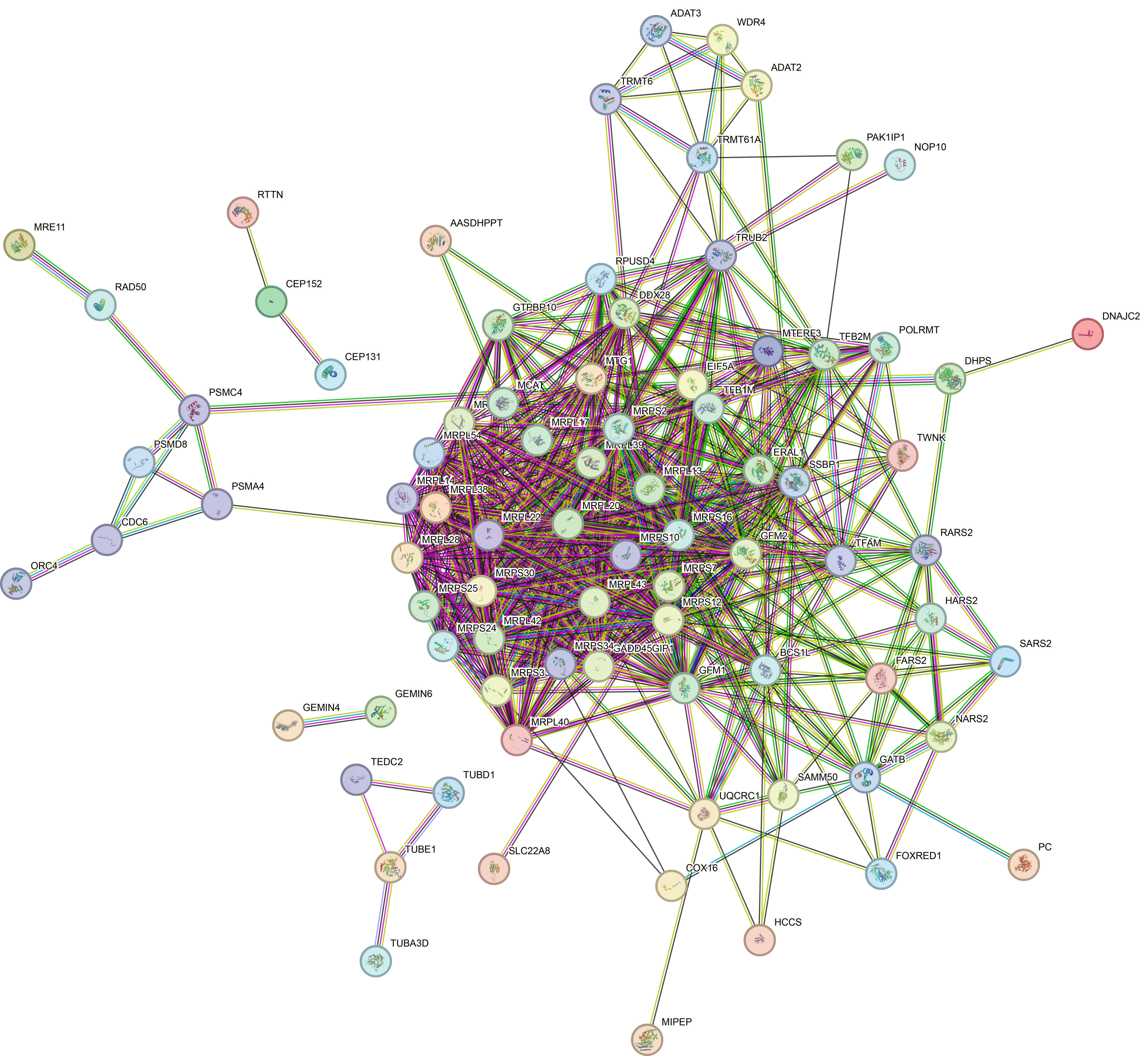

Supplement: Supplementary file 2 — Fig. S2 The details of protein-protein interaction network of FTH1-related genes. [file 262_2023_3625_MOESM2_ESM.jpg]

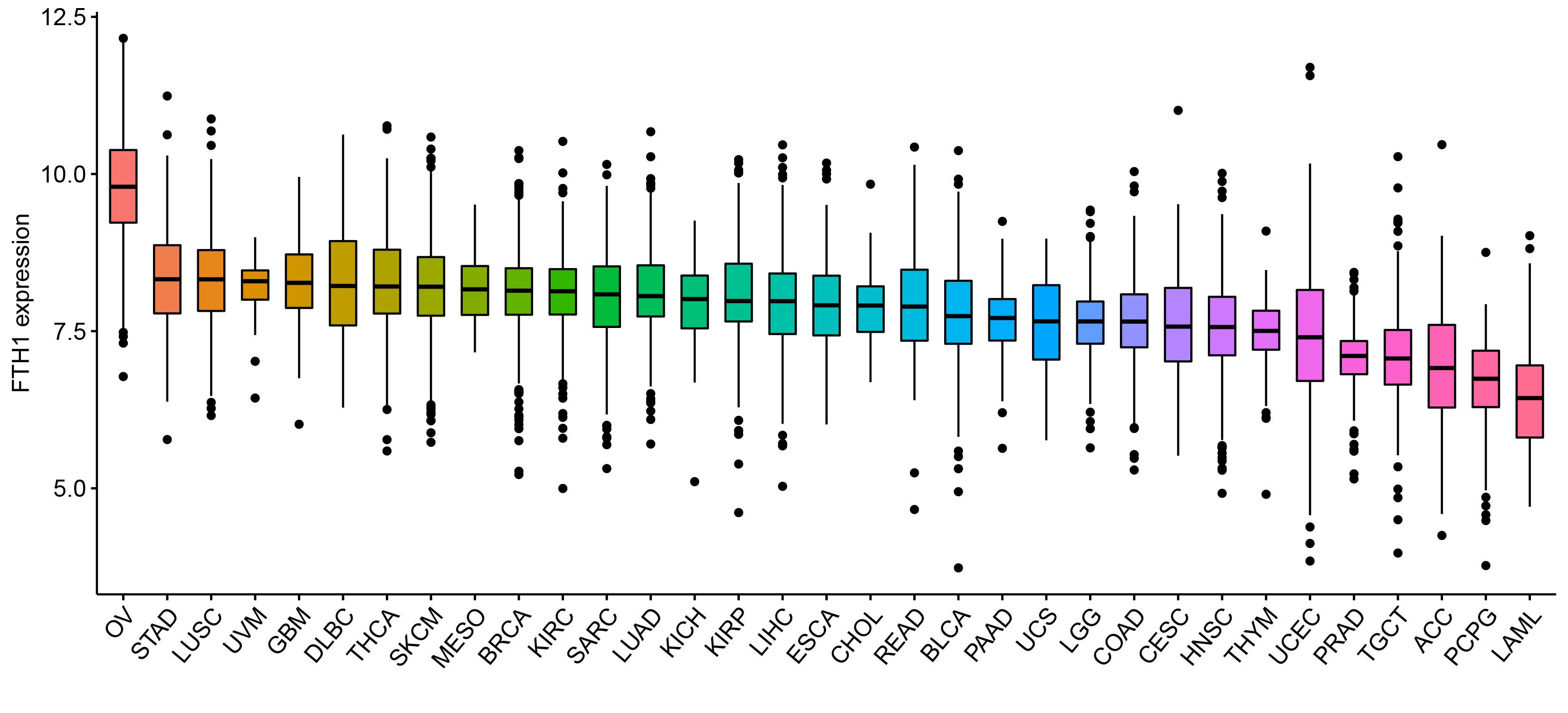

Supplement: Supplementary file 3 — Fig. S3 The mean expression of FTH1 in different cancers (from high to low). [file 262_2023_3625_MOESM3_ESM.jpg]

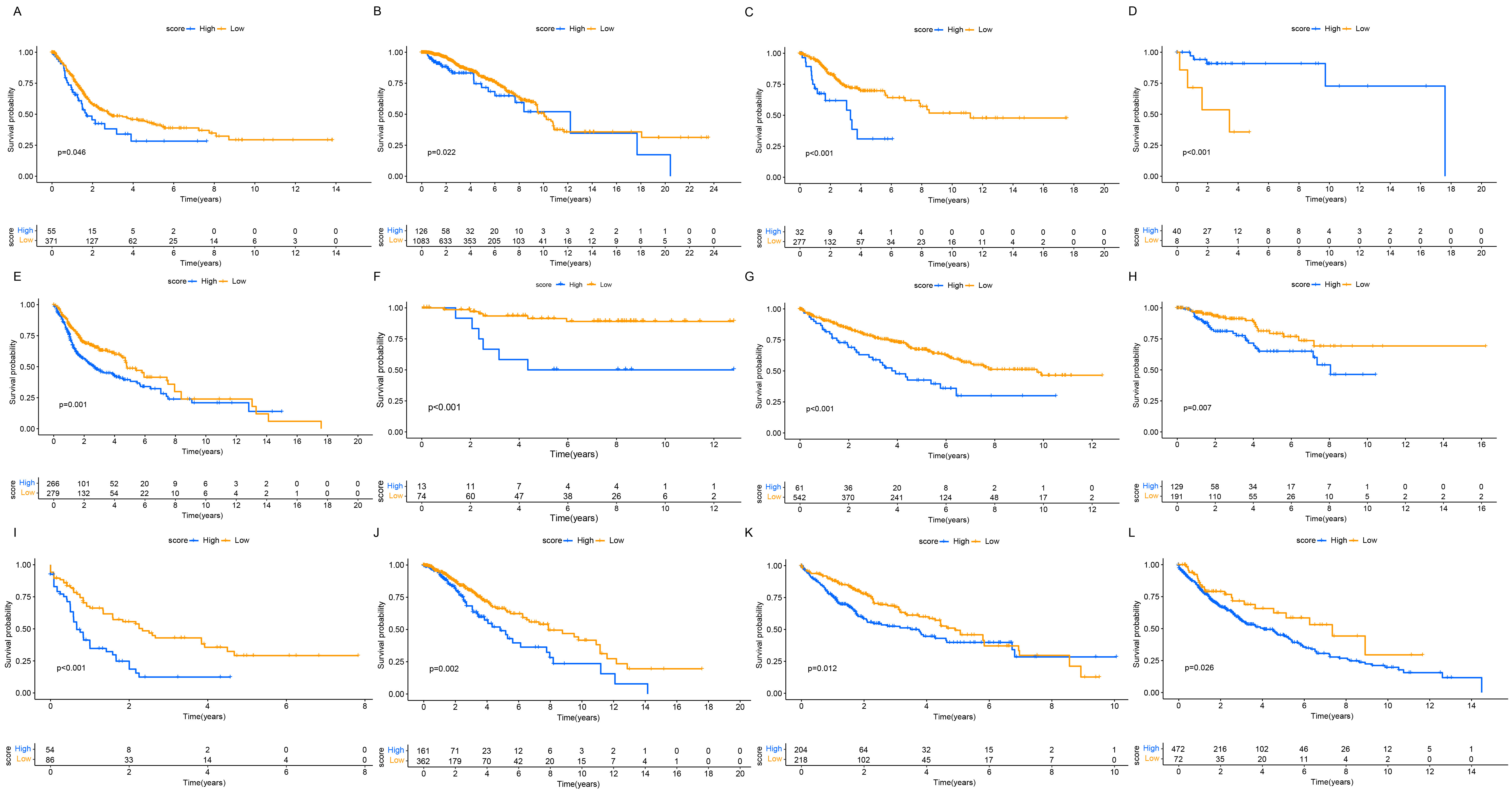

Supplement: Supplementary file 4 — Fig. S4 The Kaplan-Meier curves of OS in BLCA(A), BRCA (B), CESC (C), DLBC (D), HNSC (E), KICH (F), KIRC (G), KIRP (H), LAML (I), LGG (J), LIHC (K), and LUSC (L). [file 262_2023_3625_MOESM4_ESM.jpg]

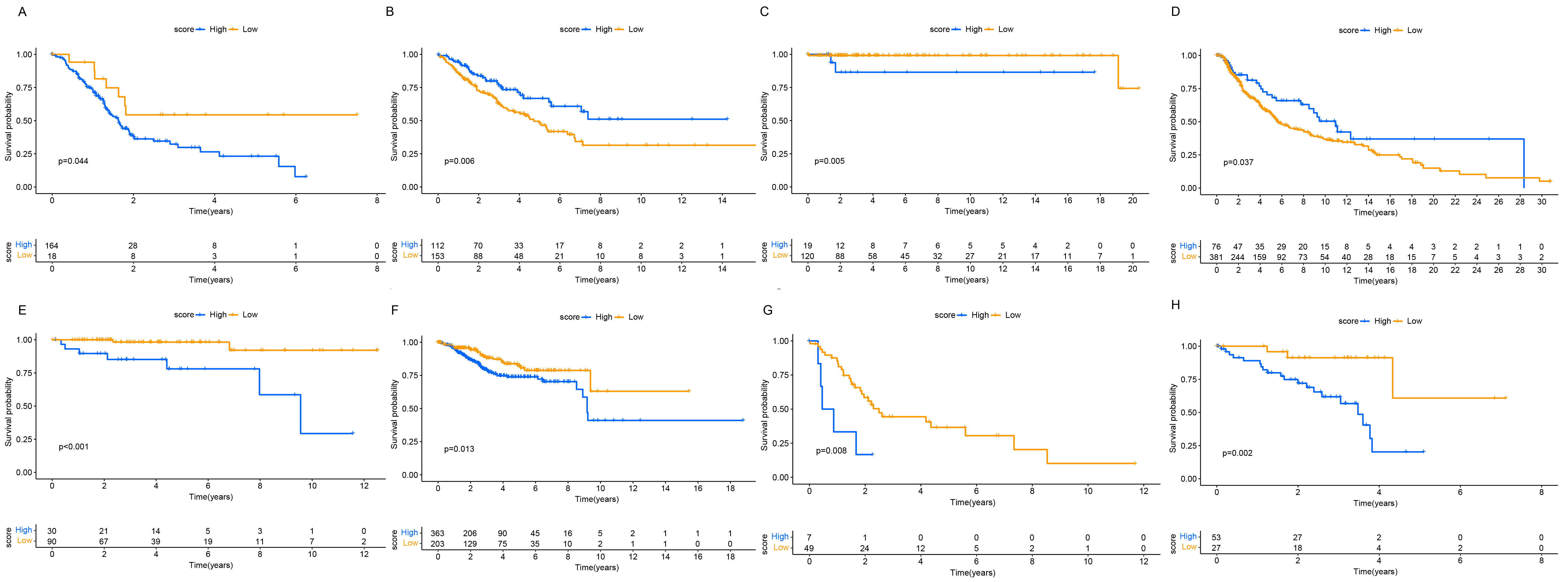

Supplement: Supplementary file 5 — Fig. S5 The Kaplan-Meier curves of OS in PAAD (A), SARC (B), SKCM (C), TGCT (D), THYM (E), UCEC (F), UCS (G), and UVM (H). [file 262_2023_3625_MOESM5_ESM.jpg]

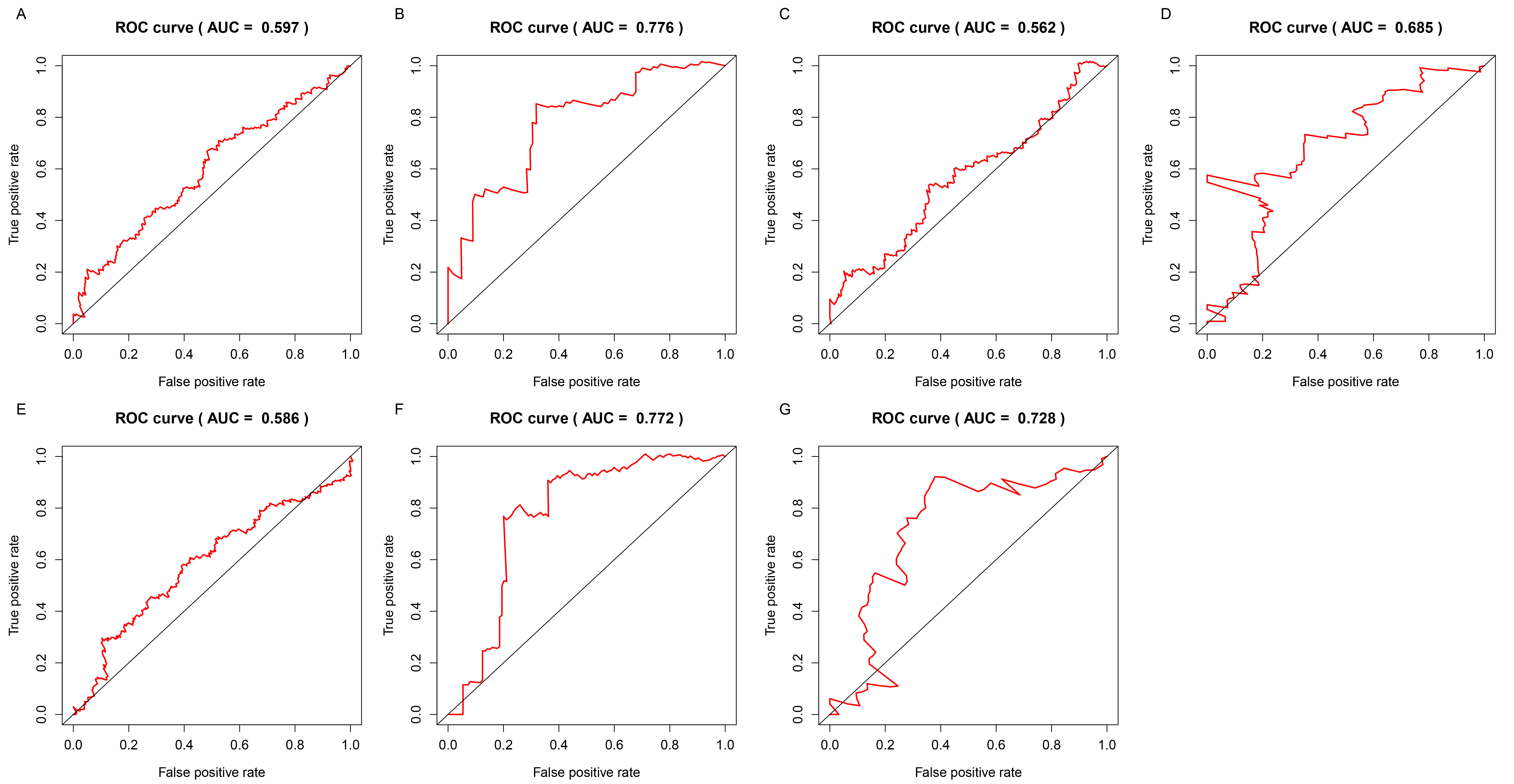

Supplement: Supplementary file 6 — Fig. S6 ROC analysis of FTH1 expression for 5-year OS in CSEC (A), KICH (B), KIRP (C), LAML (D), LGG (E), THYM (F), and UVM (G). [file 262_2023_3625_MOESM6_ESM.jpg]
